# Effectiveness of the iVac System Compared to Conventional Irrigation and Ultrasonic Activation in Reducing Microbial Biofilm, Lipopolysaccharides and Apical Extrusion

**DOI:** 10.1111/aej.12973

**Published:** 2025-07-28

**Authors:** Brenda P. F. A. Gomes, Ana B. S. Lopes, Emelly Aveiro, Lidiane M. Louzada, Ederaldo P. Godoi‐Junior, Pedro I. G. Fagundes, Esdras G. Alves‐Silva, Antônio A. L. Moura‐Filho, Rodrigo Arruda‐Vasconcelos, Juliana D. Bronzato

**Affiliations:** ^1^ Division of Endodontics, Department of Restorative Dentistry Piracicaba Dental School, State University of Campinas – UNICAMP Piracicaba SP Brazil; ^2^ Department of Restorative Dentistry School of Dentistry, University of São Paulo – USP São Paulo SP Brazil

**Keywords:** apical negative pressure, endodontics, microbiology, root canal irrigants, ultrasonics

## Abstract

This in vitro study evaluated the effectiveness of irrigation techniques (IT)—conventional irrigation (CI), ultrasonic activation (UA), and iVac system (IA)‐ using 2.5% NaOCl and saline in reducing *Enterococcus faecalis* , *Escherichia coli* and *Candida albicans* in root canals and intratubular dentine. It also assessed the reduction of lipopolysaccharides (LPS) and apical extrusion. Lower premolar roots were contaminated and divided based on IT and irrigants, with saline as control. Microbiological and LPS samples were collected before and after IT. The apical extrusion volume was determined. Results showed that when NaOCl was used, there was no statistical difference between the ITs regarding CFU reduction and between UA and IA regarding LPS reduction. When saline was used, IA was the most effective technique in reducing CFU and LPS. Regarding apical extrusion, IA caused the lowest irrigant extrusion. In conclusion, IA reduced the levels of LPS, microorganisms and apical extrusion.

## 1 Introduction

Although many technological advances exist, mechanical instrumentation and irrigation protocols cannot completely clean and disinfect root canal systems [1]. This is mainly due to the complex nature of root canals, as the anatomy prevents the irrigant from dissolving organic tissues and destroying biofilms [2]. Therefore, to improve cleaning efficiency, many activation devices are being used. These methods agitate and enhance the flow of irrigants to the complexities of the root canal system through mechanical or other forms of energy [2, 3, 4, 5].

Ultrasonic systems are one of the most used alternatives for cleaning root canal systems [4]. This activation consists of a file or flat tip oscillating at ultrasonic frequencies in a flooded canal [2, 4]. The tip transmits energy to the irrigant, producing acoustic microflow and transient cavitation, generating shear stresses that disrupt and dissociate bacterial biofilm and debris on the root canal walls [6]. It can be used continuously or intermittently [2]. However, this method can transport the irrigant beyond the distance the instrument acts, compromising the procedure's safety with the extrusion of NaOCl in the periapical tissues [4, 7].

Hence, devices using apical negative pressure have been developed (i.e., EndoVac, iVac). EndoVac extrudes less irrigant to the periapical region and avoids the formation of vapour lock [2]; however, it does not agitate the irrigants. iVac (Pac‐Dent, Brea, CA, USA) combines ultrasonic activation (UA) of the irrigant with an apical negative pressure system. In addition, iVac uses a flexible polymer cannula that allows its use in curved root canals.

Based on this, the aims of the study were: (a) to evaluate the effectiveness of irrigation techniques (IT) [conventional (CI), UA and activation with the iVac system (IA)] using 2.5% sodium hypochlorite (NaOCl) liquid and saline solution, in the reduction of *Enterococcus faecalis* , *Escherichia coli* and *Candida albicans* in the root canal and intratubular dentine; (b) to evaluate the reduction of lipopolysaccharides (LPS) and (c) to evaluate the apical extrusion by the different IT.

## 2 Material and Methods

**FIGURE 1:**
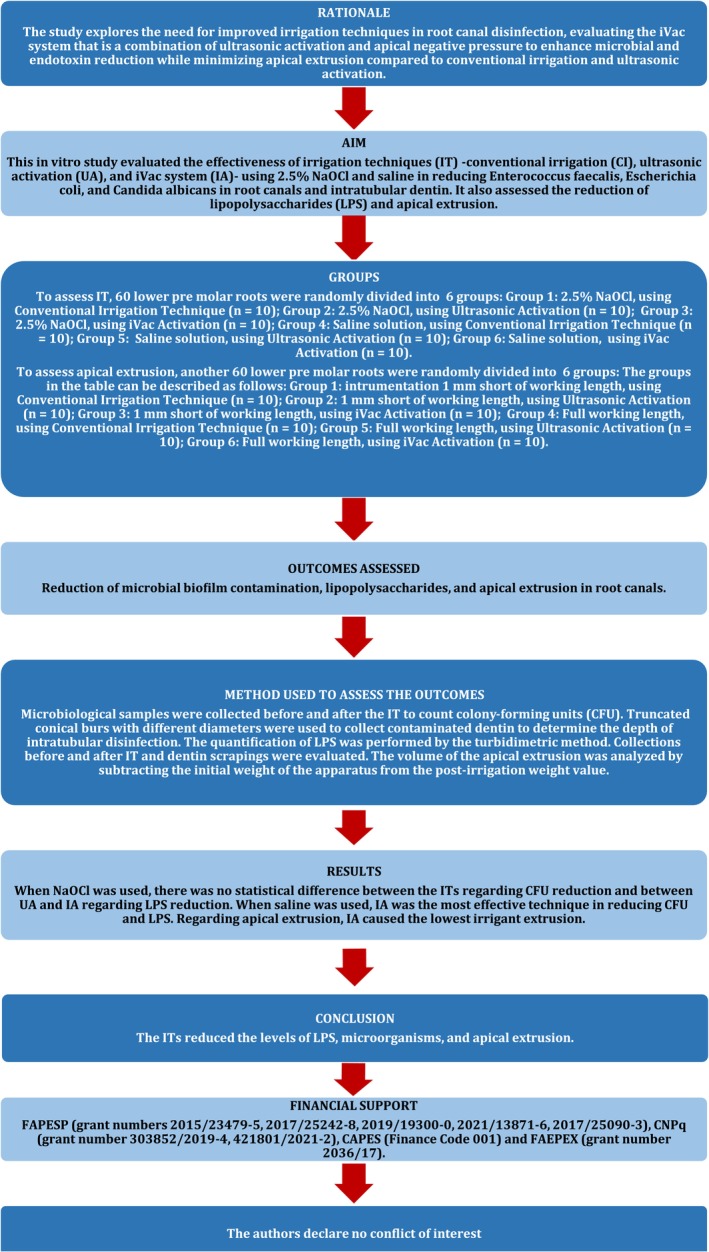
PRILE flowchart.

The manuscript of this laboratory study has been written according to Preferred Reporting Items for Laboratory studies in Endodontology (PRILE) 2021 guidelines (Figure 1). This study was approved by the Human Research Ethics Committee of the Piracicaba Dental School of the State University of Campinas, Brazil (protocol number 63728522.0.0000.5418).

### 2.1 Selected Teeth and Preparation

Selected teeth were radiographed and labelled with a digital sensor in the mesiobuccal and buccolingual planes to verify the curvature degree. Inclusion criteria involved lower premolar with a maximum curvature of 5° according to the Schneider classification [8], single‐rooted teeth with a single canal according to the Vertucci classification [9, 10], teeth with a complete apex, and foramen diameter of no more than 0.15 mm [11]. Endodontically treated teeth, internal or external resorption, root caries, fissures and/or calcifications were excluded from the research.

The dental crowns were removed with a precision cutter ISOMET 1000 (Buehler, Chicago, Illinois, USA). The roots were standardised to a length of 15 mm. The canals were progressively instrumented up to the #20 K‐file (Dentsply Sirona, Ballaigues, Switzerland) and R40 (40.06) (VDW, Munich, Germany), 1 mm short of the apical foramen. During this preparation, 1.0 mL of sterile saline was used as an irrigant at each file change. The teeth were kept sterile and moistened with gauze throughout the instrumentation to avoid dehydration.

Histological cassettes were used to divide the teeth according to their label for tooth washing.

After the initial instrumentation, the smear layer was removed by washing the teeth in 17% EDTA for 10 min, then in 5.25% NaOCl for another 10 min under constant agitation (FANEM heater‐stirrer, São Paulo, SP, Brazil). Then, the specimens were rewashed with a phosphate‐buffered solution for 10 min, followed by distilled water for 1 h to remove possible residues of EDTA and NaOCl [12, 13].

The next step was to autoclave them for 30 min at 121°C and 1 atm in screw‐capped glass vials containing 1 mL of BHI. Finally, after autoclaving, the flasks were kept for 48 h in a 10% CO_2_ incubator at 37°C to verify their sterility [14].

### 2.2 Specimens' Contamination With *Enterococcus faecalis* , *Escherichia coli* and *Candida albicans*

#### 2.2.1 Microorganisms

In a laminar flow chamber, after the growth of the *Candida albicans* (ATCC 18804), *Enterococcus faecalis* (ATCC 29212) and *Escherichia coli* (ATCC 25922) in Petri dishes and confirmation of the purity of the colony, suspensions of each microorganism were prepared separately in a sterile and pyrogenic saline solution containing 10^6^ cells/mL, which were standardised in a spectrophotometer, using a wavelength of 530 nm, 760 nm and 590 nm and optical density of 0.284, 0.298 and 0.324 for *C. albicans* , *E. faecalis* and *E. coli* , respectively.

#### 2.2.2 Contamination of 28 Days

Contamination was performed using different centrifuge cycles for 28 days. In the first 7 days, the microbial inoculum of *E. coli* (Gram‐negative) was introduced, and on the eighth day of contamination, *E. faecalis* (Gram‐positive) and *C. albicans (*fungi).

On the first day, previously standardised specimens were placed in microtubes, and 800 μL of sterile BHI broth was added to each tube. An ultrasonic bath was performed for 15 min to allow greater penetration of the culture medium in dentinal tubules before contamination. Next, the BHI broth was removed, and 800 μL of the bacterial inoculum was added to the sample tubes and centrifuged at 1400, 2000, 3600, and 5600 *g* for 2 cycles of 5 min each. After each centrifugation, the inoculum was discarded and a new one was added. After all centrifugation cycles, specimens were incubated at 37°C for 24 h.

On the second day, the tubes containing the specimens were shaken (Vortex MA 162, Marconi, Piracicaba, SP, Brazil) for 10 s, and the inoculum was discarded. Then, 1 mL of BHI sterile broth was added, and a centrifugation cycle at 3600 *g* was performed for 5 min at 25°C. Again, there was incubation in CO_2_ at 37°C for 24 h.

Starting on Day 3 and every other day until Day 21, the centrifugation procedures from the first contamination day were repeated, with inoculum added to the specimens. On the alternate days (from Day 4 to Day 20), the procedures from the second contamination day were repeated, adding only sterile BHI broth to the specimens.

**FIGURE 2:**
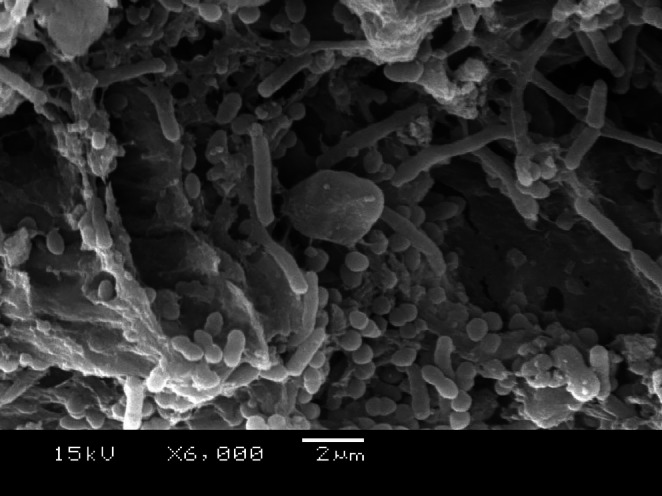
Scanning Electron Microscopy micrograph of contamination of dentinal tubules (6000×).

Finally, from the 22nd day, 20 μL of sterile BHI broth was added inside the root canals every 2 days, without using the centrifuge, totalling 28 days. Confirmation of dentinal tubule contamination was observed through scanning electron microscopy (SEM) (Figure 2). Then, the collection procedures described below were performed.

### 2.3 Experimental Groups

To prevent the extrusion of irrigants through the apices, all teeth had their apex sealed with wax, which was disinfected with 5% NaOCl and subsequently neutralised with 5% sodium thiosulfate (Drogal Pharmaceuticals Ltda., Piracicaba, SP, Brazil). After that, the 60 roots were randomly divided into six groups (Table 1), according to the auxiliary chemical substances (2.5% NaOCl solution or saline solution—Drogal Pharmaceuticals Ltda., Piracicaba, SP, Brazil) and the IT used (conventional, ultrasonic, and iVac). Groups 4, 5, and 6 were used as controls. In Table 2, there is a description of volumes and sequences of irrigating substances used in all IT tested.

**TABLE 1:** —Specimen distribution (*n*) by group according to the tested auxiliary chemical substances and irrigation techniques.

Groups	Irrigant	Neutralizer	Techniques	Specimens (n)
1	2.5% NaOCl	5% Sodium thiosulfate	Conventional	10
2	2.5% NaOCl	5% Sodium thiosulfate	Ultrasonic Activation	10
3	2.5% NaOCl	5% Sodium thiosulfate	iVac Activation	10
4	Saline solution	—	Conventional	10
5	Saline solution	—	Ultrasonic Activation	10
6	Saline solution	—	iVac Activation	10

**TABLE 2:** Description of volumes and sequences of irrigating substances used in all irrigation techniques tested.

	Conventional technique	Ultrasonic activation	iVac activation
Groups 1, 2 e 3	2.5% NaOCl (30 mL) 5% Sodium thiosulfate (5 mL) Distilled water (15 mL) 17% EDTA (30 mL) Distilled water (15 mL) 2.5% NaOCl (30 mL) 5% Sodium thiosulfate (5 mL) Distilled water (15 mL)	2.5% NaOCl (30 mL) 5% Sodium thiosulfate (5 mL) Distilled water (15 mL) 17% EDTA (30 mL) Distilled water (15 mL) 2.5% NaOCl (30 mL) 5% Sodium thiosulfate (5 mL) Distilled water (15 mL)	2.5% NaOCl (30 mL) 5% Sodium thiosulfate (5 mL) Distilled water (15 mL) 17% EDTA (30 mL) Distilled water (15 mL) 2.5% NaOCl (30 mL) 5% Sodium thiosulfate (5 mL) Distilled water (15 mL)
Groups 4, 5 e 6^a^	Saline solution (30 mL) Saline (5 mL) Distilled water (15 mL) 17% EDTA (30 mL) Distilled water (15 mL) Saline (30 mL) Saline (5 mL) Distilled water (15 mL)	Saline solution (30 mL) Saline (5 mL) Distilled water (15 mL) 17% EDTA (30 mL) Distilled water (15 mL) Saline (30 mL) Saline (5 mL) Distilled water (15 mL)	Saline solution (30 mL) Saline (5 mL) Distilled water (15 mL) 17% EDTA (30 mL) Distilled water (15 mL) Saline (30 mL) Saline (5 mL) Distilled water (15 mL)

^a^ Saline instead 5% sodium thiosulfate and 2.5% NaOCl.

#### 2.3.1 Irrigation Techniques

##### 2.3.1.1 Conventional Technique

All irrigation was performed with a 5 mL syringe and 30‐gauge NaviTip needle (Ultradent, South Jordan, USA) placed 2 mm short from the apex using in and out movements and with constant suction.

##### 2.3.1.2 Ultrasonic Activation

All irrigation was performed with a 5 mL syringe and a 30‐gauge NaviTip needle (Ultradent, South Jordan, USA) placed 2 mm from the apex without exerting apical pressure. When the NaOCl or saline solution was used, they were subjected to UA for 1 min (3 cycles of 20 s), with an activation every 10 mL of solution dispensed. The E1 ultrasonic tip (Helse Dental Technology, Santa Rosa de Viterbo, SP, Brazil) was inserted 2 mm from the root canal length [13], and the power of the ultrasonic device was 30% (Sonus, Medidenta, Las Vegas, NV, USA) according to the manufacturer's instructions. In order to set up the best power control of the ultrasound, it was necessary to find the point of fog and water dripping because excessive power can cause a risk of tip fracture [14]. E1 (also called Irrisonic) is a metallic insert with a diameter of 20.01 [15]. There was constant suction with the sucker placed at the root canal entrance.

##### 2.3.1.3 iVac Activation

**FIGURE 3:**
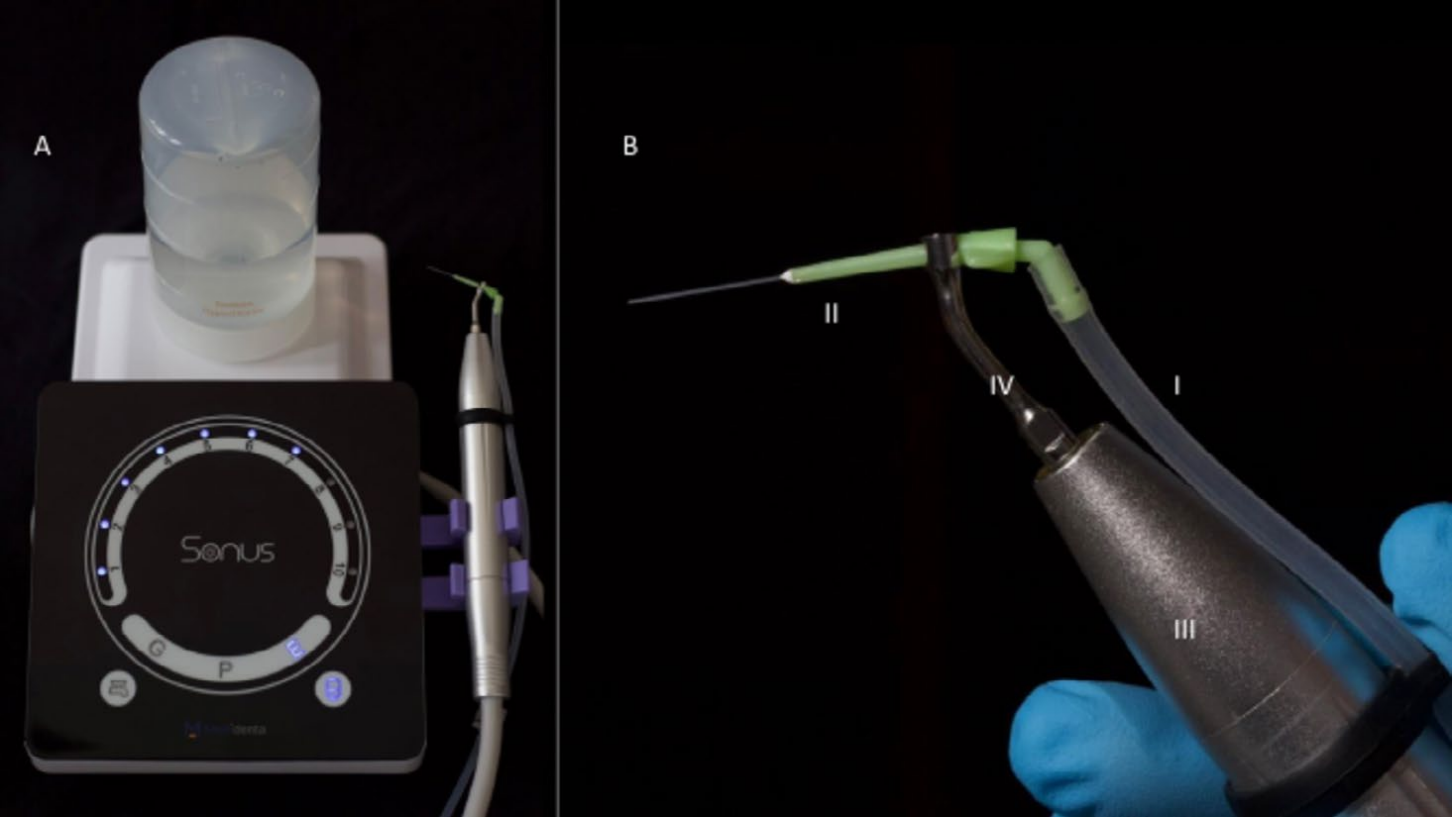
iVac device. (A) Sonus piezoelectric ultrasonic unit with iVac system attached. (B) 0.35‐mm cannula (II) connected to small suction tubes (I) attached to a piezoelectric ultrasonic handpiece (III) through a connector (IV) (S‐type insert).

The iVac 0.35 tip was attached to a piezoelectric ultrasonic handpiece (Sonus, Medidenta, Las Vegas, NV) through a connector (iVac S‐type) (Figure 3). Next, an elbow‐type connection is coupled to the rear opening of the cannula, which connects to an evacuation tube. The iVac ultrasonic connector is designed to hold the cannula and deliver the irrigating liquid while transmitting the vibration from the piezo ultrasonic handpiece. In addition, tubes and connections allow the ordinary vacuum outlet to be coupled, adding negative pressure to the system (Figure 3).

All irrigation was performed with iVac 0.35 tips (with an internal diameter of 0.15 mm) inserted 1 mm from the root canal length (at working length), and the power of the ultrasonic device was 70% according to the manufacturer's instructions. Also, care was taken to avoid physical contact of the ultrasonic and iVac tips to the root canal walls [13].

### 2.4 Initial and Final Bacterial/LPS Sampling

After contamination and before the irrigation procedure started, the teeth were placed on a sterile metal platform inside the laminar flow chamber. Each root was irrigated with 1.0 mL of sterile saline there, and microbiological and LPS initial samples were collected from the contaminated root canal with sterile absorbent paper cones # 20 (TANARI, Tanarian Industrial Ltda, Macapurú, AM, Brazil). Then these cones were deposited individually in sterile Eppendorf tubes (Elkay Products Inc., Shrewsbury, MA, USA) containing 1 mL of BHI.

The irrigation procedure (described above) was done, and after the final irrigation with a neutraliser or sterile saline, according to each group, the canals were dried with sterile absorbent paper cones, which were deposited in individual microtubes containing 1 mL of BHI. This was considered the final collection.

### 2.5 Dentinal Shavings Collection

This methodology was previously described [16]. Each specimen was fixed on sterile aluminium support for the sequential removal of dentine shavings from the surface of the canal lumen that was in contact with the irrigants tested during instrumentation. For this, sterile diamond truncated conical burs with an increasing diameter were used in the sequence as follows: 3069 (Drill 1/ISO 018), whose largest diameter is 1.8 mm, 3139 (Drill 2/ISO 021), whose largest diameter is 2.1 mm and 4137 (Drill 3/ISO 025) whose largest diameter is 2.5 mm, at low speed. The burs were placed in and out of the canal three times. The dentine shavings obtained using each bur were immediately collected in glass vials containing 1.0 mL of sterile BHI.

### 2.6 Microbiological Sample Preparation (Dilution, Counting of Colony‐Forming Units)

Immediately after each collection, the microbiological samples (initial and final collection, and dentinal shavings collection) were shaken (Vortex MA 162, Marconi, Piracicaba, SP, Brazil) for 1 min and diluted in series 1/10, 1/100, 1/1000 and 1/10000 in sterile BHI. Twenty‐five microliters of the dilutions and the original solution were plated in triplicates on BHI agar with 5% sheep blood and incubated in a CO_2_ incubator at 37°C for 48 h. After incubation, the colonies of microorganisms on the plates were counted by determining the colony‐forming units (CFU).

### 2.7 Quantification of LPS—LAL Pyrogent 5000

LPS was quantified in the initial and final LPS samples collected using paper points and in the dentine shavings samples [17]. The LAL chromogenic test (Lonza, Basel, Switzerland) was utilised following the established protocol described in previous studies, in accordance with the manufacturer's instructions [18].

### 2.8 Apical Extrusion

Another sixty lower premolars were selected and included as described in the first section of material and methods. The tooth crown was also cut as previously described; however, the instrumentation with reciprocating files (Reciproc R40, VDW, Munich, Germany) was performed following two different protocols (Table 3): (a) in which the first was performed 1 mm short of the root canal full length (14 mm), (b) while the second method was performed at the root canal full length corresponding to the foramen exit (15 mm). After the instrumentation, the apical extrusion test was performed.

**TABLE 3:** Description of specimens (*n*) by irrigation technique and working length.

Groups	Work length	Techniques	Specimens (*n*)
1	1 mm short	Conventional	10
2	1 mm short	Ultrasonic activation	10
3	1 mm short	iVac activation	10
4	Full length	Conventional	10
5	Full length	Ultrasonic activation	10
6	Full length	iVac activation	10

This test aimed to evaluate the extrusion of the NaOCl in all IT used here (Table 3). The lower premolars' roots were covered with a Teflon band, except for the last 1 mm of the apical third, and inserted into a hole made in the cap of the Eppendorf tube. Subsequently, the interface between the tube cap and the tooth was hermetically sealed with cyanoacrylate and covered with a top dam (FGM, Joinville, SC, Brazil) to prevent fluid leakage into the tube during the preparation of the tube [19, 20].

A needle was inserted in the tube cap and the Eppendorf tubes were placed in a glass bottle using a rubber dam to hold them in place. The rubber dam did not allow the operator to see the root during endodontic preparation, avoiding bias. Finally, the apparatus was labelled and weighed three times on a 10^−4^ high‐precision electronic balance (NOVAK, São José do Rio Preto, SP, Brazil). The mean value was calculated (weight 1).

Then, the lower premolars' roots were submitted to the different techniques (conventional, ultrasonic and iVac with 2.5% NaOCl) as described above in Section 2.3.1.

After performing the different IT, the same apparatus was weighed three times, and its average value was calculated (weight 2). Finally, the value of the initial weight (weight 1) was subtracted from the final weight (weight 2) to quantify the extrusion of the irrigants.

## 3 Statistical Analysis

Regarding CFU reduction in root canal and intratubular dentine and LPS differences, a three‐way factorial analysis of variance (ANOVA) was applied for overall comparisons. In addition, the Tukey HSD test was applied for pairwise comparisons. Regarding apical extrusion data, a paired Student *t*‐test was used. The level of significance adopted was 5%.

## 4 Results

### 4.1 CFU Reduction in Root Canal

Significant CFU reduction was observed across all treatments, with notable differences based on chemical substance, time, and agitation method (CI, UA, IA) (*p* < 0.0001). For NaOCl + EDTA, CFU reduction was 100%, so comparisons focused on saline + EDTA. UA and IA groups showed similar reductions (*p* = 0.957), both outperforming CI (*p* < 0.0001) (Table 4).

**TABLE 4:** CFU mean, standard deviation and reduction percentage before and after different treatment protocols.

Group	Saline solution + EDTA			NaOCl + EDTA		
	Before	After	Reduction percentage	Before	After	Reduction percentage
CI	1 280 000 (±201 935)	65 200 (±12 951)	94.75 a	1 858 000 (±324 749)	0	100
UA	1 435 000 (±39 511)	2920 (±850)	99.80 b	1 389 000 (±44 833)	0	100
IA	1 583 000 (±97 985)	1500 (±956)	99.91 b	2 164 000 (±199 288)	0	100

*Note:* Three‐way factorial analysis of variance, followed by Tukey HSD. Different letters mean a statistical difference between the agitation methods regarding each chemical substance separately.

Abbreviations: CI, Conventional irrigation; iVac activation; UA, ultrasonic activation.

### 4.2 CFU Reduction in Intratubular Dentine

Statistically significant CFU reductions were observed for chemical substance, bur type, and agitation method (*p* < 0.0001). As with root canal results, NaOCl+EDTA yielded zero CFU, so comparisons were made using saline+EDTA. For each bur, IA achieved the highest CFU reduction, followed by UA, then CI (*p* < 0.0001) (Table 5).

**TABLE 5:** CFU mean (standard deviation) according to the method of agitation and bur (B).

	Saline+EDTA		
	B1	B2	B3
CI	1508 (188.67) a	1175 (200.35) a	997 (170.49) a
UA	920 (204.4) b	675 (207.16) b	355 (68.52) b
IA	320 (147.57) c	130 (125.17) c	50 (70.71) c

*Note:* Three‐way factorial analysis of variance, followed by Tukey HSD. Different letters mean a statistical difference between the agitation methods and bur.

Abbreviations: B1, 3069 bur; B2, 3139 bur; B3, 4137 bur ; CI, Conventional irrigation; IA, iVac Activation, UA, ultrasonic activation.

### 4.3 LPS Quantification

LPS reduction varied significantly across chemical substances, time, and agitation methods (*p* < 0.0001). For saline+EDTA, all agitation methods differed significantly (*p* < 0.01). With NaOCl+EDTA, no differences were observed between UA and IA (*p* = 0.933), but both outperformed CI (*p* < 0.0001) (Table 6).

**TABLE 6:** LPS levels (EU/mL) mean and standard deviation in log10, and percentage reduction before and after different treatment protocols.

Group	Saline solution + EDTA			NaOCl + EDTA		
	Before	After	Percentage reduction	Before	After	Percentage reduction
CI	3.76 (±0.06)	2.01 (±0.03)	98.23 a	3.63 (±0.13)	1.72 (±0.03)	98.77 a
UA	3.67 (±0.04)	1.76 (±0.07)	98.7 b	3.67 (±0.06)	0.99 (±0.06)	99.79 b
IA	3.63 (±0.11)	1.51 (±0.03)	99.24 c	3.69 (±0.02)	0.88 (±0.15)	99.84 b

*Note:* Three‐way factorial analysis of variance, followed by Tukey HSD. Different letters mean a statistical difference between the agitation methods regarding each chemical substance separately.

Abbreviations: CI, Conventional irrigation; iVac activation; UA, ultrasonic activation.

### 4.4 Apical Extrusion Analysis

Irrigant extrusion varied significantly by agitation method (CI > UA > IA) for both −1 mm and 0 mm limits (*p* < 0.05). No significant differences in extrusion volumes were found between limits for any method (*p* > 0.05) (Table 7).

**TABLE 7:** Mean and standard deviation (SD) regarding the extrusion of irrigants after irrigation with conventional irrigation (CI), ultrasonic activation (UA) and iVac activation (IA) in the instrumentation limit at −1 and 0 in grams.

Instrumentation limit	Conventional irrigation (CI)		Ultrasonic activation (UA)		iVac activation (IA)	
	Mean	SD	Mean	SD	Mean	SD
Apex −1 mm (−1)	0.516^Aa^	0.380	0.166^Ba^	0.042	0.016^Ca^	0.004
Apex (0)	0.545^Aa^	0.121	0.202^Ba^	0.051	0.016^Ca^	0.004

*Note:* Paired student *t*‐test. Different capital letters indicate the presence of a statistical difference between the lines. Distinct lowercase letters represent the presence of a statistical difference between columns.

## 5 Discussion

### 5.1 CFU Reduction in Root Canal

In this study, a multispecies biofilm comprising Gram‐positive bacteria ( *Enterococcus faecalis* ), Gram‐negative bacteria ( *Escherichia coli* ) and a fungus ( *Candida albicans* ) was employed to more accurately simulate the polymicrobial nature of the root canal environment. The root canal system is characterised predominantly by bacterial species but also includes fungi, archaea, and viruses, reflecting a complex microbiota [21, 22].

The inclusion of *E. faecalis* is particularly relevant due to its well documented association with persistent endodontic infections and treatment failures, attributed in part to its ability to invade dentinal tubules and adhere to collagen via the antigen I/II family of polypeptides [23, 24, 25]. Moreover, this microorganism is capable of forming biofilms which provide protection against the host's immune defences and hinder the effectiveness of antibiotics, making their complete eradication a significant clinical challenge [26].

*Escherichia coli* represents a Gram‐negative facultative bacterium characterised by the presence of lipopolysaccharide (LPS) molecules anchored in its outer membrane. Although it is not frequently isolated from root canal infections, *E. coli* and its LPS are commonly employed in studies evaluating LPS disinfection protocols. This is largely due to several factors: (a) *E. coli* serves as the gold standard in LPS research and produces one of the most biologically active LPS types; (b) its LPS is the reference material used in limulus amebocyte lysate (LAL) assays for LPS quantification and (c) it is widely available in commercial form, facilitating standardised experimentation [27].

The inclusion of *Candida albicans* addresses the fungal component known to colonise the root canal system and contribute to persistent infections [28]. In combination, these selected microorganisms establish a more comprehensive and clinically relevant model of the root canal microbial ecosystem, particularly given their well documented resistance to antimicrobial strategies. Consequently, their reduction or elimination can serve as a meaningful indicator of the efficacy of a given disinfection protocol.

When analysing the reduction of CFU in the root canal, there was no statistically significant difference between the irrigation methods when using NaOCl (Table 4). After all, sodium hypochlorite is considered the most potent disinfectant in endodontics due to its excellent ability to dissolve vital and necrotic tissues and its antimicrobial activity [29]. Furthermore, the effectiveness of sodium hypochlorite can be improved by increasing the volume of the irrigant [30]. This experiment used a large volume of irrigants, totaling 60 mL of NaOCl. Also, this result can be explained by the fact that it used a biofilm model with only three species in canals without calcification and curvatures. Future research should test a more complex biofilm model in curved root canals where irrigation with agitation may show a more significant result, being close to the clinical situation [29].

The use of 60 mL of NaOCl in this study is justified by the specific characteristics of the piezoelectric ultrasonic device (Sonus, Medidenta, Las Vegas, NV), which incorporates three separate liquid reservoirs. This design necessitates a higher volume of irrigant to maintain continuous flow and maximise disinfection efficiency within shorter time intervals. Similarly, other advanced irrigation systems, such as the GentleWave System, require substantial irrigant volumes, delivering up to 45 mL per minute while simultaneously applying negative‐pressure irrigation. In other words, 3 or 5 min of irrigation with NaOCl is equivalent to 135 or 225 mL [31]. These findings highlight that systems designed to optimise cleaning and disinfection often rely on larger irrigant volumes to achieve their intended effects.

However, in clinical practice, the volume of NaOCl used depends on the irrigation technique and the equipment available. For clinicians using an ultrasonic device without built‐in reservoirs—such as when pairing an ultrasonic unit with the IvacSystem—irrigation is performed manually with a syringe. In these cases, the total irrigant volume is influenced by the number of instruments used during root canal preparation.

A commonly recommended protocol involves using 5 mL of NaOCl before the introduction of each file, followed by 5 mL of EDTA and 5 mL of distilled water, leading to a total volume of at least 35 mL [13]. Although this volume is lower than that required by ultrasonic devices with separate liquid reservoirs, it remains within clinically accepted standards. In this study, the same volume of irrigant was delivered to all groups to avoid bias. It is important to emphasise that increasing the volume of irrigant contributes to enhanced disinfection by improving the mechanical flushing of debris, penetration into dentinal tubules and overall antimicrobial effectiveness.

Therefore, it was only possible to visualise a statistical difference when the saline solution was used, as it does not have antimicrobial action. A statistical difference was observed when methods that activated the saline solution (UA and IA) were used, but with no difference. This occurred because the mechanical action of the movement caused by the UA of the saline solution removes significantly more bacteria than conventional irrigation with a syringe. However, the saline solution is not bactericidal and does not dissolve the organic tissue [32].

The use of distilled water as an intermediate irrigant was based on its ability to prevent unwanted chemical interactions between chemical substances [33]. Although this approach is not universally adopted as a standard protocol for alternating irrigants, distilled water is widely used as a final rinse after EDTA [13]. Scientific evidence supports the role of an inert solution in effectively flushing out residual irrigants within the root canal system [33]. This way, distilled water was used in all groups to avoid bias.

### 5.2 CFU Reduction in Intratubular Dentine

As in the analysis of the reduction of CFU in the root canal, when NaOCl was used, the percentage of CFU was zero for all groups (Table 5). Therefore, it was only possible to notice a statistically significant difference between the agitation methods when saline was used as an irrigating substance. A more significant CFU reduction was observed in the iVac group, followed by UA and the conventional irrigation group.

The iVac system uses continuous ultrasonic‐activated irrigation. Although both continuous ultrasonic irrigation and intermittent UA represent types of ultrasonic‐assisted irrigation, their fluid dynamics and antibiofilm efficacy differed, probably due to different application modes. One desired effect of ultrasonic‐activated irrigation is acoustic microstreaming, which critically depends on filling the canal with liquid. Continuous UA involves continuous replenishment of irrigant, keeping the canal filled with irrigant. In contrast, intermittent is applied to a given volume of irrigant within the canal. Due to a directional flow of irrigant from the ultrasonic tip coronally, the irrigant volume is depleted during activation, which may impede acoustic microstreaming [34].

A previous study [4] evaluated a device similar to the iVac, which also uses a continuous apical negative ultrasonic irrigation (CANUI); however, it is composed of a nickel‐titanium microcannula. CANUI improved irrigant penetration into the lateral canals and up to the working length. This corroborates with our results, where it was observed that a better reduction in the number of CFU in all thirds and depths when using the iVac system.

### 5.3 LPS Quantification

If, after endodontic treatment, some residual endotoxin reaches the periradicular tissues, it modulates the host immune responses by the expression of cytokines and metalloproteinases [35]. Therefore, we should consider using auxiliary methods to reduce endotoxin levels in root canals as much as possible [17].

There was a statistically significant difference between the chemical substances, the time, and the agitation methods (Table 6). In addition to NaOCl, EDTA was also associated with a widely used chelator for removing the contaminated smear layer and the debris formed during mechanical chemical preparation. EDTA acts in the deep layers (≈130 μm) of the contaminated dentine not reached by the preparation. In addition, it can potentiate the removal of LPS due to its potential to bind to the calcium present in the lipid A portion of the endotoxin molecules [17].

Few studies analysed the reduction of LPS levels with different activation systems. Our results agree with previous research [17], which obtained a significant improvement in the removal of endotoxins after UA of EDTA but without being able to eliminate them. Another study [36] also showed that PUI improves NaOCl action over *E. faecalis* , *E. coli* and their endotoxins.

Nevertheless, in other studies [37, 38] there was no statistical difference between conventional irrigation and UA. However, there were some differences in the methodology, that is, a higher concentration of sodium hypochlorite (6%) was used, which enhanced the neutralisation of LPS [37].

### 5.4 Apical Extrusion

In all groups, there was some volume of irrigant extruded, as described in a previous study [39]. They noted that irrigant extrusion seems inevitable unless negative pressure irrigation is used [39]. Although the present experiment used a simple in vitro model [15], the results are in accordance with the literature, including a systematic review that revealed that apical negative pressure irrigation (ANP) prevents apical extrusion of irrigants [40]. Future studies should compare iVac with other negative pressure systems.

### 5.5 Final Considerations

To mitigate the impact of confounders, it was implemented a rigorous experimental design that included randomisation of specimens into groups, standardisation of root canal preparation protocols, and controlled environmental conditions during microbial contamination and irrigation procedures. In terms of statistical analysis, it was employed a three‐way factorial ANOVA to account for interactions between the key variables: irrigation technique, chemical substance, and sampling time. The use of Tukey's HSD post hoc test ensured that multiple comparisons were statistically controlled, reducing the risk of Type I errors. Assumptions were verified before performing statistical tests, ensuring the robustness of our conclusions. Implementing these measures was aimed to enhance the internal validity of our study and ensure that the observed differences between groups were attributable to the tested IT rather than external variables.

Based on the findings of this in vitro study, clinicians are encouraged to consider the use of continuous apical negative‐pressure ultrasonic irrigation as part of their standard disinfection protocols, particularly in cases with complex canal anatomy or increased risk of irrigant extrusion. When combined with conventional irrigants, such as 2.5% sodium hypochlorite and 17% EDTA, this approach may enhance full length canal disinfection, minimise apical extrusion and achieve greater intratubular microbial reduction, potentially leading to improved periapical healing and reduced incidence of post‐operative flare‐ups. Future research should include randomised clinical trials evaluating microbial reduction, patient‐reported outcomes (e.g., post‐operative pain and periapical healing) and cost‐effectiveness across different tooth types, canal anatomies and irrigant concentrations. Additional studies are also needed to determine optimal activation durations and to employ high‐resolution imaging techniques for in situ assessment of irrigant penetration and biofilm disruption. Addressing these translational aspects will enhance evidence‐based recommendations and help to fully realise the clinical potential of negative‐pressure irrigation.

## 6 Conclusion

In conclusion, IA reduced the levels of LPS, microorganisms, and apical extrusion.

## Author Contributions

Conceptualization: Brenda P. F. A. Gomes. Methodology: Brenda P. F. A. Gomes, Ana B. S. Lopes, Emelly Aveiro, Lidiane M. Louzada, Ederaldo P. Godoi‐Junior, Pedro I. G. Fagundes, Esdras G. Alves‐Silva, Antônio A. L. Moura‐Filho, Rodrigo Arruda‐Vasconcelos, Juliana D. Bronzato. Software: not applicable. Validation: Brenda P. F. A. Gomes, Ana B. S. Lopes, Emelly Aveiro, Lidiane M. Louzada, Ederaldo P. Godoi‐Junior, Pedro I. G. Fagundes, Esdras G. Alves‐Silva, Antônio A. L. Moura‐Filho, Rodrigo Arruda‐Vasconcelos, Juliana D. Bronzato. Formal analysis: Brenda P. F. A. Gomes, Ana B. S. Lopes, Emelly Aveiro, Ederaldo P. Godoi‐Junior, Juliana D. Bronzato. Investigation: Brenda P. F. A. Gomes, Ana B. S. Lopes, Emelly Aveiro, Lidiane M. Louzada, Ederaldo P. Godoi‐Junior, Pedro I. G. Fagundes, Esdras G. Alves‐Silva, Antônio A. L. Moura‐Filho, Rodrigo Arruda‐Vasconcelos, Juliana D. Bronzato. Resources: Brenda P. F. A. Gomes. Data curation: Brenda P. F. A. Gomes. Writing – original draft: Brenda P. F. A. Gomes, Juliana D. Bronzato, Emelly de Aveiro. Writing – review and editing preparation: Brenda P. F. A. Gomes, Juliana D. Bronzato. Visualisation: Brenda P. F. A. Gomes. Supervision: Brenda P. F. A. Gomes. Project administration: Brenda P. F. A. Gomes. Funding acquisition: Brenda P. F. A. Gomes.

## Disclosure

All authors have contributed significantly, and all authors are in agreement with the manuscript.

## Conflicts of Interest

The authors declare no conflicts of interest.

## Data Availability

The data that support the findings of this study are available from the corresponding author upon reasonable request.
